# Pain Management through Neurocognitive Therapeutic Exercises in Hypermobile Ehlers–Danlos Syndrome Patients with Chronic Low Back Pain

**DOI:** 10.1155/2021/6664864

**Published:** 2021-06-01

**Authors:** Claudia Celletti, Teresa Paolucci, Loredana Maggi, Giordana Volpi, Mariangela Billi, Roberta Mollica, Filippo Camerota

**Affiliations:** ^1^Physical Medicine and Rehabilitation Division, Umberto I University Hospital of Rome, Italy; ^2^Unit of Physical Medicine and Rehabilitation, G. D' Annunzio University of Chieti-Pescara, Department of Oral Medical Science and Biotechnology (DSMOB), Chieti, Italy; ^3^Physical Medicine and Rehabilitation, Department of Geriatrics, Neurosciences and Orthopedics, Catholic University of the Sacred Heart, 00100 Rome, Italy

## Abstract

**Background:**

The hypermobile type of Ehlers–Danlos syndrome (hEDS) is likely the most common hereditary disorder of connective tissue mainly characterized by joint hypermobility. Patients with hEDS suffer joint pain, in particular low back pain, commonly resistant to drug therapy. The aim of this research was to evaluate a neurocognitive rehabilitation approach based not only on the motion and function recovery but also on the pain management.

**Methods:**

In this nonrandomized clinical trial, eighteen hEDS patients (4 males and 14 females) with mean age 21 years (range 13-55) were recruited and evaluated before and after three months of rehabilitation treatment.

**Results:**

The outcome scores showed significant statistical results after treatment in reducing pain symptoms (numerical rating scale, *P* = 0.003; McGill (total score), *P* = 0.03), fatigue (fatigue severity scale, *P* = 0.03), fear of movement (Tampa scale, *P* = 0.003), and pain-associated disability (Oswestry disability index, *P* = 0.03).

**Conclusion:**

The clinical results observed in our study seem to confirm the role of a specific neurocognitive rehabilitation program in the chronic pain management in the Ehlers–Danlos syndrome; the rehabilitation treatment should be tailored on patient problems and focused not only in the recovery of movement but also on pain perception.

## 1. Introduction

Hypermobility Ehlers–Danlos syndrome is a heritable connective tissue disorder recently described among the 13 subtypes of Ehlers–Danlos syndrome [1]. The hypermobile type of Ehlers–Danlos syndrome (hEDS) (previously known as EDS type III according to the Berlin nosology [2] and EDS hypermobility type according to Villefranche nosology [3]) is likely the most common hereditary disorder of connective tissue primarily identified as having generalized joint hypermobility, related musculoskeletal connective tissue disorder manifestations, and a milder involvement of the skin, which lacks the degree of cutaneous features typically observed in the classical and vascular types of EDS [1–4]. Since the Villefranche nosology, the clinical description of hEDS in the medical literature has expanded considerably to include more features, such as chronic pain, chronic fatigue, dysautonomia, and anxiety among other associated symptoms. Patients with hEDS usually suffer joint pains, in particular low back pain that commonly is resistant to drug therapy. They frequently have a poor posture characterized by lumbar hyperlordosis and dorsal hyperkyphosis. The current research suggests that combining education and exercise with cognitive behavioral approaches may be particularly effective for improving pain and reducing disability in adolescents and adults [5–8]. Then, considering these premises, the aim of this research as a pilot study was to evaluate a neurocognitive rehabilitation approach based on the pain management and reduction as primary outcome.

## 2. Materials and Methods

### 2.1. Population

All patients were consecutively enrolled in the outpatient rehabilitation unit for hypermobility disorders of the Umberto I University Polyclinic Hospital of Rome, after a physiatrist evaluation. Inclusion criteria were as follows: diagnosis of hypermobile EDS according to the 2017 International Classification of Ehlers–Danlos Syndromes [1] done by a clinical geneticist and chronic low back pain (LBP). Patients with postsurgical pain condition or with a low back pain secondary to surgical treatment were excluded. Patients have been recruited from March 2017 to February 2018; only two patients refused to participate for personal and working reasons. This study was performed as per the Helsinki Declaration on human experimentation and was approved by the Local Institutional Review Board REF. CE 4789. All patients gave written informed consent after receiving detailed information on the study's aims and procedures.

This study referred to TREND guidelines for clinical nonrandomized study.

### 2.2. Rehabilitative Plan

The rehabilitative treatment was performed for one time a week (sixty minutes for each session) for three consecutive months. All the patients continued the daily common activities and ongoing drug therapy. The scope of the treatment has been to reduce and treat the main aspect characterizing the patients: the reduced muscle recruitment, the chronic fatigue, the fear of movement, and the recovery of an adequate motor strategy, in particular of the back spine, but always focused on the patients' feeling of perception of pain and the functional limitations with respect to the principles of the narrative medicine [9].

During the treatment, the neurocognitive approach followed different phases.

“*Felt sense*” approach or feeling felt: it is necessary to direct the bodily sensations initially focusing the attention on the healthy parts of the body and then describing the painful typology, localization, and perception in order to confine it on the low back pain region. This first 10- to 15-minute phase was based on the language and narration of lower back pain and its characteristics. For example, the patient was asked to sense the pleasant contact (comfortable warmth) of the therapist's (PT) hands as slight pressure with the palm to the region of the back without pain. Afterward, the PT asked the patient to bind the pleasant and pain-free sensation to the painful area.

The PT used language, through the approach of narrative medicine [9, 10], asking the patient how the sensation of contact with the painful area changed during the session, what were the sensations that he/she received (of pleasure and relief or of discomfort). The physiotherapist guided with his/her voice and hands the perception of contact when necessary. The objective of the neurocognitive rehabilitation proposals, starting from learning how to correctly perceive the painful part through the felt sense, was to help the patient learn how to control the pathological elements that led to the establishment and evolution of inadequate compensation and persistence of pain.

Helping the patient to research physiological and nonpainful strategies to learn the most physiological possible movements and to rebuild a bodily image consistent with reality, using the spatial orientation of limbs, using the touch and the pressure discrimination, in particular along the trunk midline, this part lasted for the remaining 45 minutes of the rehabilitation session.

### 2.3. Exercises and Rehabilitative Protocol

**Figure 1:**
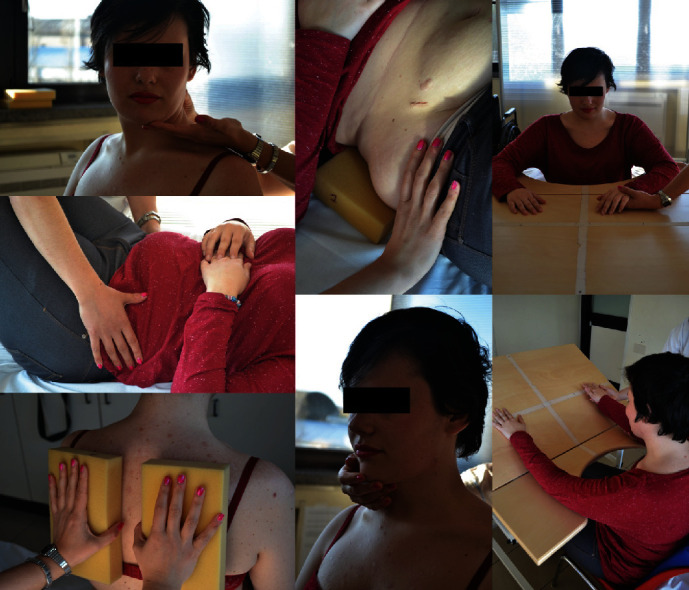
An example of progressive neurocognitive exercises (cautious mobilization of the cervical spine, exercise of lumbopelvic mobilization, exercise of recognition of the sponges, and exercises of recognition and coordination of the position of the upper limbs with respect to the reference of the midline).

1. Recognition of the location of the contact, at the trunk level. Modality: the patient sitting (if it is not possible, use the supine position), contact with the therapist's hand is employed, using different requests (=tasks) and methods (=passive or active modality, with eyes open or closed), or with sponges of different consistency. Starting from the area of the shoulder blades then the lumbar and the features of the spine, it considered first with the less painful region and therefore more accessible to contact. The recognition of the contact occurs through complete contact with the physiotherapist's hand or only with the fingertips, as much depending on the proprioceptive discrimination that the patient has to solve; the PT could request and exercised contact with full hand or only with the fingertips. Also, the therapist's hand can lightly touch, fully touch, or exert light pressure (Figure 1). An example of request (=task) was recognition of sponges of different consistency, placed in correspondence of the interscapular space, or the medial edge of the scapula, or the spine of the scapula, or the clavicle and the coracoacromial arch. Methods: the exercise was proposed initially in passive mode with eyes closed, and when a sufficient muscle relaxation was obtained, the exercise was proposed in active mode (it was not the PT that pushed the sponges against the scapular region, but the patient tried to push on the sponges) first with eyes closed and then with opened eyes. Preferably, the sponges were applied symmetrically. Three textures were considered: hard, medium hard, and soft depending on the compressibility and springback. The shape of the sponges, square or rectangular, with a width comfortable to be contained by the palm of the physiotherapist's hand, had a variable height from 5 to 10 centimeters
2. Recognition of positions through the lumbopelvic rhythm. Modality: both with the patient seated and on his back, through diaphragmatic breathing, his attention can be directed to the lumbopelvic movement and to small modifications
3. Recognition of two sponges of different consistency at the pelvis level, through the lumbopelvic rhythm. Modality: the patient was in supine position and lower limbs quite flexed or seated in symmetrical position, feet placed on floor, hips, knees, and ankles flexed at 90°, upper limbs at their sides. The *required task was to* identify the location, size, and consistency of different sponges placed at the level of the lumbar region. These exercises were executed with eyes closed
4. Recognition of the tilting of the pelvis and of the laterolateral and anteroposterior relationship of the trunk. Modality: the patient is seated in symmetrical position, feet placed on the floor, hips, knees, and ankles flexed at 90°, upper limbs at their sides, pelvis placed on a board able to rock in all directions through a gimbal (a mechanism, typically consisting of rings pivoted at right angles, for keeping the two planes parallel or inclined relative to each other as needed) [10]. *Modality*: identification of thicknesses of different heights and of diverse resistance springs placed under the aid. The exercise was executed first with eyes closed and then with eyes open. The exercise was conducted to research the postural symmetry perception of the spine considering the trunk midline
5. Recognition of the symmetrization of the load. Modality: the patient is in standing position, back resting against a wall, feet parallel, and lower limbs placed on two sets of scales to control the organization of load. Planned laterolateral transferal of load. Subsequently, the same task can be requested, in third grade, with the trunk detached from the wall. Each of these rehabilitative sections lasted about 10-15 minutes. This rehabilitation protocol, which is based on neurocognitive rehabilitation, has taken up some exercises proposed in the treatment of chronic back pain and adapted and customized to hEDS patients

### 2.4. Outcomes

All patients have been clinically evaluated before and after treatment with different clinical outcome scales: McGill pain questionnaire (MG) [11], the Tampa scale (TSK) [12, 13], the fatigue severity scale (FSS) [14], the Oswestry disability index (ODI) [15], and the numerical rating scale (NRS) [16] for pain.

The McGill pain questionnaire is probably the most well-known and complete tool for the verbal assessment of pain. It provides a subjective measurement of pain intensity as well as clues on qualitative features of the chronic or acute pain experienced by the patients. Results broadly fall into 3 main classes: (1) sensory qualities (temporal, spatial, thermal, pressure, and other qualities); (2) affective qualities (tension, fear, and autonomic properties); and (3) intensity of pain. Descriptors are also subdivided into 20 subclasses and arranged in increasing order of pain intensity [11].

TSK is the most widely used questionnaire to assess pain and pain-related fear of movement in subjects with musculoskeletal complaints, and this has been translated into and validated in different languages including Italian [12]. Kinesiophobia is frequent and related to pain and fatigue in hEDS patients [11]. TSK is divided into two subscales: evaluating activity avoidance and harm, respectively. TSK is able to distinguish the fear of movement domain from other conceptual domains such as pain and functional alteration. The original version of the TSK-I questionnaire comprises 17 items to assess the subjective rating of kinesiophobia [12, 13]. FSS is a scale quantifying fatigue intensity, which has been used in different chronic conditions, such as multiple sclerosis and systemic lupus erythematosus, and shows high internal consistency and validity. FSS comprises 9 items with a 7-point response format that indicates the degree of agreement with each statement [14].

The ODI consists of ten questions pertaining to daily activities and covers the following: experiencing general pain, practicing self-care (e.g., washing and dressing), lifting objects, sitting, standing, walking, sleeping, travelling, engaging in sexual activity if applicable, and participating in social activities. The items are rated on 6-point scales scored in the range of 0–5, with higher scores indicating higher pain-associated disability [15].

NRS is a rapid-to-administrate 11-point numerical scale used to roughly measure any kind of pain with a score ranging from 0 (no pain) to 10 (acute pain) [16].

Moreover, the proportion of patients achieving a higher than a reduction of approximately two points of the NRS pain scores from baseline as minimal clinically important difference (MCID) has been calculated [17], in association with a percentage of patients with a reduction of 10 points percentage in ODI scale as MCID [18].

### 2.5. Statistical Analysis

Statistical analysis was conducted with the SPSS software package for Windows, version 19.0. The statistical analysis of the continuous variables was conducted calculating median and range (min–max), because these variables were not normally distributed. In order to evaluate the functional scales adopted, we used the Wilcoxon test for paired samples. The significance level was set at *P* < 0.05.

## 3. Results

### 3.1. Patients' Descriptive Data

Eighteen patients (4 males and 14 females, mean age 21 years, min 13, max 55) have been recruited for this study. All the patients have followed the complete period of treatment except for two that have missed one day appointment for personal reasons. All patients referred no changes in daily activities.

### 3.2. Outcomes

Results of the functional scales before and after rehabilitative treatment are illustrated in Table 1.

**Figure 2:**
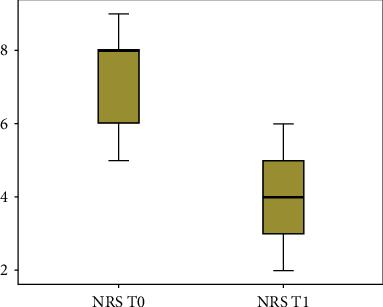
The box plot diagram of numerical rating scale (NRS) evaluated at T0 (before) and T1 (after treatment). The spacings between the different parts of the box indicate the degree of dispersion, the minimum, first quartile, median, third quartile, and maximum values.

**Figure 3:**
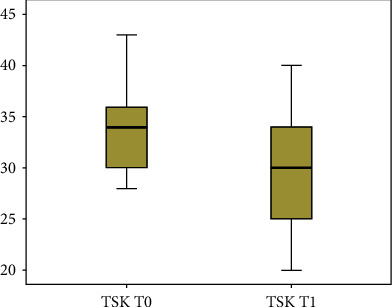
The box plot diagram of Tampa scale (TSK) evaluated at T0 (before) and T1 (after treatment).

Patient outcome scores showed significant results after treatment in reducing pain symptoms, fatigue, kinesiophobia, and pain-associated disability as illustrated in Figures 2 and 3 using the box plot diagram; after treatment, the results showed an improvement in lower back pain by NRS (*P* = 0.003) and McGill (total score) (*P* = 0.03), a reduction in fatigue by FSS (*P* = 0.03), a better attitude to move with less fear by Tampa scale (*P* = 0.003), and, last but not least, an improvement in pain-associated disability by Oswestry disability index (*P* = 0.03). Considering the MCID for pain, the incidence of a significant score variation was 88.9% while for the ODI scale it was 42%.

## 4. Discussion

Pain is a common symptom referred as complex and disabling in patients with hEDS [15]. A different hypothesis has been evaluated in the last years, and the underlying mechanisms are still unclear. Some patients suffer joint-related pain, but in most of them, pain has a complex distribution poorly compatible with joint-related pain. Also, symptoms may induce to evaluate the neuropathic characteristics of pain [19], but electrophysiologically, the somatosensory nervous system is not damaged [20, 21].

Recently, another hypothesis has been suggested to explain the mechanisms of pain. In particular, an incongruence between sensory input and central motor output (i.e., sensory-motor incongruence) reflecting discrepancies between the motor and sensory cortex may lead to generalized and unfocused pain in some hEDS patients. This phenomenon, hypothesized in other chronic pain conditions, implies defective sensory input coupled with disinhibition of the motor output [21].

More specifically, Di Stefano et al. [22] have studied hEDS patients with quantitative sensory testing methods that included thermal pain perceptive thresholds and the wind-up ratio. They showed no somatosensory nervous system damage associated to an increased wind-up ratio that implies that pain in hEDS might arise through central sensitization. According to Woolf [23], central sensitization is “operationally defined as an amplification of neural signaling within the central nervous system that elicits pain hypersensitivity” and “augmented responsiveness of central nervous system neurons to their normal or subthreshold afferent input” [24]. Central sensitization (CS) reflects increased activity of pain facilitation pathways and malfunctioning of descending pain inhibitory pathways which result in dysfunctional endogenous analgesic control. In addition, the pain neuromatrix is likely to be overactive in patients with CS [25, 26]. Compared to those classified as having peripheral neuropathic pain and nociceptive pain, patients with CS reported more severe pain, poorer general health-related quality of life, and greater levels of back pain-related disability, depression, and anxiety.

The clinical results observed in our study seem to suggest the role of a specific neurocognitive rehabilitation program in the chronic low back pain management; the rehabilitation treatment should be tailored on patient problems and focused not only in the recovery of movement but also focalize on pain perception. Also, if this is only a pilot study and no electrophysiological evaluation has been done (like for example QST and CPM), it is the first neurocognitive rehabilitation attempt in hEDS and the results are encouraging, not only with respect to the improvement and reduction of chronic LBP in this specific pathology but also—and above all—because an approach based on movement awareness has also allowed to reduce fear linked to the movement itself. In the treatment of chronic pain, neurocognitive rehabilitation has shown good results in the literature and the use of the motor imagery is proving successful [27–30]. hEDS patients have a fear of movement, and moreover, a very intense rehabilitation approach is not indicated and does not help in improving chronic pain. The neurocognitive rehabilitation approach has been tested with good results in chronic pain, and its characteristics, according to our hypothesis, could be adapted to hEDS patients with chronic LBP.

An organizing relationship of the trunk that rapidly disappears in the patient with chronic pain is the flexibility and fragmentability of the movement itself, with a reduction in the quality and quantity of movement, the maintenance of fixed postures that are not adequate for function. Using the neurocognitive exercise, through the exteroceptive and perceptive inputs, the recovery of movement is obtained through the resolution of a cognitive task (as can be the recognition exercise with sponges of different consistency or weight shift, as described in the specific section of the paper). Then, the patient is placed in front of a perceptive task, which therefore requires the integrity of cognitive processes, such as attention and the ability to process tactile, proprioceptive, pressure, kinesthetic information, as well as the ability to interact actively with the contact surface. Unlike the traditional rehabilitation approach, the neurocognitive approach is based on specific rehabilitation settings that could help the patient to extract meaningful information that can lead to conscious recovery improving what that we defined “felt sense.” In fact, the three principles of neurocognitive therapeutic exercises are to assume (i) rehabilitation as a learning process, (ii) the body as a receptor surface, and (iii) the movement as knowledge. Then, each proposed exercise had to be broken down and respect the “*control*” with regard to the movement parameters (i.e., spatiality, temporality, and intensity) [31–33]. Also, motor imagery (MI) in neurocognitive rehabilitation continues to provide an impetus for new findings relating to our emotional network, embodied cognition, inhibitory processes, and action representation. The evocation of a correct MI would allow a greater coherence in the body self, causing then the pain relief. It should also be noted that the evocation of a correct MI, necessary to increase the ability to gain correct somesthesic information and thus generating the chronic pain remission, has been the most difficult element to achieve during the rehabilitation process for both cases studied. The MI thus provides a means at the service of thinking and learning, as it allows simulating and anticipating, guiding and facilitating the perception. Also, Moseley studied as when pain becomes chronic, the efficacy of the pain neuromatrix is strengthened via nociceptive and nonnociceptive mechanisms, which means that less input, both nociceptive and nonnociceptive, is required to produce pain: rehabilitation progresses to increase exposure to threatening input across sensory and nonsensory domains [8, 34, 35]. Moreover, biopsychosocial treatment which acknowledges and addresses the biological, psychological, and social contributions to pain and disability is currently seen as the most efficacious approach to chronic pain [6–8].

## 5. Conclusions

These clinical results seem to confirm the role of a specific neurocognitive rehabilitation program in the chronic pain management in the Ehlers–Danlos syndrome, effective in reducing low back pain and the related disability. An important aspect, contained in our proposed neurocognitive rehabilitation program, is that the rehabilitation treatment should be tailored on patient problems and focused not only in the recovery of movement but also on pain perception for better rehabilitative responses. At last, this study has some limitations such as the lack of a control group, a limited sample size, the evaluation done with only clinical scales, and the absence of a functional evaluation of patients like spine segmental mobility function. Then, our results, although encouraging, should be interpreted with caution. Our data should be integrated with studies applied on a big cohort of patients associated with clinical and neurophysiological evaluation in order to confirm this pilot rehabilitative novel proposal.

## Figures and Tables

**Table 1:** Results of the scales administrated before (T0) and after (T1) treatment.

Clinical scales	T0	T1	^∗^ *P* < 0.05
FSS	46 (32-60)	40 (23-58)	**≤0.05**
TSK	34 (28-43)	30 (20-40)	**≤0.001**
NRS	8 (5-9)	4 (2-6)	**≤0.001**
ODI	16 (5-35)	10 (3-29)	**≤0.001**
McGill (total score)	45 (26-55)	37 (16-46)	**≤0.001**
MG sensitive subscore	26 (16-31)	20 (9-29)	**≤0.001**
MG affective subscore	7 (3-12)	5 (2-8)	**≤0.001**
MG cognitive subscore	4 (2-5)	3 (1-4)	**≤0.002**

^∗^Significant *P* values are in bold. FSS = fatigue severity scale; TSK = Tampa scale; NRS = numerical rating scale; ODI = Oswestry disability index.

## Data Availability

The data can be available upon request from the corresponding author.
